# Roles of Nrf2 in Gastric Cancer: Targeting for Therapeutic Strategies

**DOI:** 10.3390/molecules26113157

**Published:** 2021-05-25

**Authors:** Tahereh Farkhondeh, Ali Mohammad Pourbagher-Shahri, Mohsen Azimi-Nezhad, Fatemeh Forouzanfar, Aranka Brockmueller, Milad Ashrafizadeh, Marjan Talebi, Mehdi Shakibaei, Saeed Samarghandian

**Affiliations:** 1 Cardiovascular Diseases Research Center, Birjand University of Medical Sciences, Birjand 9717853577, Iran; farkhondeh2324@gmail.com; 2 Faculty of Pharmacy, Birjand University of Medical Sciences, Birjand 9717853577, Iran; 3 Faculty of Medicine, Birjand University of Medical Sciences, Birjand 9717853577, Iran; ali.pourbagher.shahri@gmail.com; 4 Noncommunicable Diseases Research Center, Neyshabur University of Medical Sciences, Neyshabur 9318614139, Iran; aziminm@mums.ac.ir; 5 Medical Toxicology Research Center, School of Medicine, Mashhad University of Medical Sciences, Mashhad 9177948564, Iran; forouzanfarff@gmail.com; 6 Musculoskeletal Research Group and Tumor Biology, Chair of Vegetative Anatomy, Institute of Anatomy, Faculty of Medicine, Ludwig-Maximilian-University Munich, Pettenkoferstrasse 11, D-80336 Munich, Germany; aranka.brockmueller@med.uni-muenchen.de; 7 Faculty of Engineering and Natural Sciences, Sabanci University, Orta Mahalle, Üniversite Caddesi No. 27, Orhanlı, Tuzla, Istanbul 34956, Turkey; dvm.milad73@yahoo.com; 8 Sabanci University Nanotechnology Research and Application Center (SUNUM), Tuzla, Istanbul 34956, Turkey; 9 Department of Pharmacognosy, School of Pharmacy, Shahid Beheshti University of Medical Sciences, Tehran 1996835113, Iran; talebi.m@sbmu.ac.ir

**Keywords:** gastric cancer, Nrf2 pathway, Nrf2 inhibitor, antioxidants

## Abstract

Nuclear Factor Erythroid 2-Related Factor 2 (Nrf2) is a specific transcription factor with potent effects on the regulation of antioxidant gene expression that modulates cell hemostasis under various conditions in tissues. However, the effects of Nrf2 on gastric cancer (GC) are not fully elucidated and understood. Evidence suggests that uncontrolled Nrf2 expression and activation has been observed more frequently in malignant tumors, including GC cells, which is then associated with increased antioxidant capacity, chemoresistance, and poor clinical prognosis. Moreover, Nrf2 inhibitors and the associated modulation of tumor cell redox balance have shown that Nrf2 also has beneficial effects on the therapy of various cancers, including GC. Based on previous findings on the important role of Nrf2 in GC therapy, it is of great interest to scientists in basic and clinical tumor research that Nrf2 can be active as both an oncogene and a tumor suppressor depending on different background situations.

## 1. Introduction

Gastric cancer (GC) is one of the most common malignant tumor diseases with a very high mortality rate globally [1,2,3]. Symptoms of this disease do not become apparent until the advanced stage of disease. In recent decades, a continuous decrease in the incidence rates of GC has been found worldwide [4,5,6,7], as the decrease in GC rate may be related to the improvement of hygiene and food standards and the eradication of *Helicobacter pylori* (*H. pylori*) [8]. Genetic background, environmental pollutants, cigarette smoking, alcohol consumption, socioeconomic status, and *H. pylori* infection are known to be the main risk factors for GC [9,10,11]. Other risk factors for GC include Epstein–Barr virus (EBV), pernicious anemia, blood type A, obesity, and gastrectomy [12,13,14,15,16]. To manage the treatment of GC, classifications such as early and advanced stages have been established, and anatomically, this cancer is classified into three types: distal esophagus, cardia, and stomach distal to cardia. 

The tumorigenesis and dissemination of malignant cells is modulated by the exposure of cells to detrimental or protective elements. The interaction between these factors is found at the cellular level via the strict modulation of signaling pathways for which their activation or suppression can either maintain or impair the malignant transformation of normal cells and the increased aggressiveness of cancer cells. Kelch-like ECH-associated protein 1(Keap1)–nuclear factor erythroid 2-related factor 2 (Nrf2) is the most studied signaling pathway of cellular defense against oxidative stress [12].

Nrf2 is a transcription factor that regulates the expression of genes that have antioxidant response element-like sequences in their promoter to antioxidant and reactive oxygen species (ROS) detoxification [17] such as heme oxygenase-1 (HO1), peroxiredoxins, and glutamate-cysteine ligase (GCL) [18,19,20,21,22,23]. Nrf2 protects normal cells against DNA damage induced by ROS and malignant cells against chemotherapy [24,25]. Nrf2 also stimulates several oncogenes unrelated to antioxidant activity, such as matrix metallopeptidase 9 (*MMP-9*), tumor necrosis factor *α* (*TNF-α*), and vascular endothelial growth factor A (*VEGF-A*) [23].

Several factors can modulate Nrf2 in GC cells [26,27,28,29]. Increased production of ROS disassociates the Nrf2 from Keap1, resulting in the translocation of Nrf2 to the nucleus. In addition, P62, a regulator protein, interacts with Keap1 to mediate its degradation, leading to an increase in the concentration of Nrf2 in the nucleus [30,31,32]. It has been further reported that P21 can interact with Nrf2, thereby stabilizing the Nrf2 protein [33]. Within the nucleus, the small V-Maf avian musculoaponeurotic fibrosarcoma oncogenic MAF proteins (sMAF) bind with Nrf2 to form an Nrf2/sMaf heterodimer [18]. Nrf2/sMaf heterodimers regulate the expression of target genes through binding antioxidant response elements (AREs) or MAF recognition elements (MARE) [34,35]. 

It has been reported that mutations of Nrf2 or its regulators may lead to the suppression or over-stimulation of Nrf2 signaling [36,37]. Nowadays, numerous scientific reports indicate the efficacy of targeting Nrf2 in various diseases [38]. In addition, there is evidence of the beneficial effect of Nrf2 activity and the suppressive role of Nrf2 inhibitors against tumor progression [39,40,41] in human cancers [42,43,44]. However, contradictory activity of Nrf2 in tumor progression has been found in recent years [39,40,41,42,45,46]. In a cancer cell, the overactivation of antioxidant defenses, in particular, Nrf2-dependent genes following ROS production, increases the ability to survive under oxidative and inflammatory stress conditions. It is mostly dependent on the expression of several genes with different antioxidant and cytoprotective activities induced by Nrf2 [9]. Various Nrf2 target genes are related to cancer cell proliferation and death, such as genes involved in the pentose phosphate pathway and responsible for NADPH and purine regeneration, leading to the stimulation of cancer cell proliferation [36,37,38]. HO-1 is another target of Nrf2 that has potent antioxidant and anti-apoptotic effects that can increase cancer cell growth and resistance to therapy [38]. High Nrf2-HO-1 levels have mostly been found in malignant tumor cells with aggressiveness and poor outcome [38]. Indeed, tumor cells use Nrf2 as a protective mechanism to increase their survival. The paradoxical activity of Nrf2 in GC suggests a “dark side profile” in the progression of GC tumors.

Nrf2 may also induce malignancy in tumor cells by binding to the ARE sequence in the promoter region of Notch1 and the p53 inhibitor Mdm2. Mdm2 expression induces high levels of p53, leading to cell death [36,37,38]. In addition, Nrf2 induces angiogenesis through stimulating hypoxia-inducible factor 1-alpha (HIF-1*α*)-dependent vascular endothelial growth factor (VEGF) expression in cancer cells and promoting cancer growth [36,37,38].

However, the exact mechanisms for these oncogenic effects of Nrf2 in cancer development are not clear. This study reviewed the available publications on the roles of Nrf2 in GC, focusing on the Nrf2 inhibitors and activators to summarize information for easy access for future investigations on GC therapy.

## 2. Treatment Modalities of Gastric Cancer

For diagnosed gastric cancer (GC), the only curative therapeutic option is surgical resection [39]. Other therapeutic options in GC, such as perioperative and adjuvant chemotherapy along with chemoradiation, are more suitable when GC can be resected and lymph nodes need to be dissected [39]. Altogether, these strategies result in a 15% increase in survival compared with surgery alone [39].

In the classical surgical settings, for the thorough resection and staging of GC, total gastrectomy and subtotal gastrectomy along with lymphadenectomy are the common approaches [39]. For the extent of gastric resection, 2 to 6 cm gross margins are the range reported to be most appropriate for the minimal chance of microscopic positive margins. However, there are no universally acceptable extents for lymphadenectomy [47]. The emergence of minimally invasive gastrectomy using laparoscopic and robotic gastrectomy has brought advantages, such as reduced blood loss, morbidity, and hospitalization periods, and a shorter normalization of bowel function. The oncologic outcome regarding mortality and lymph node procurement are similar between open and minimally invasive surgery for GC [48]. The peritoneal spread of gastric adenocarcinoma needs different treatment modalities than surgical resection alone because of its poor prognosis and high recurrence rate.

Surgical removal of GC is feasible in only 20% of cases. Locoregional and/or metastatic recurrences reduce the median survival after surgery to 12–20 months. These problems led to the use of neoadjuvant or adjuvant treatment modalities in addition to surgery. Therapeutic strategies with adjuvant chemotherapy compared with surgery alone have shown conflicting results in terms of survival [49]. More reliable results obtained from several meta-analyses shows that adjuvant chemotherapy has a significant benefit, as it can improve the overall survival and decrease the recurrence rates compared with surgery alone [50,51,52,53].

Adjuvant chemoradiation has also shown a survival benefit for the management of GC [54]. Studies have shown an advantage for adjuvant chemoradiation compared with adjuvant radiotherapy or chemotherapy alone [55,56]. For completely resected carcinoma of the esophagogastric junction or GC, treatment with postoperative chemoradiation has been suggested as the standard of care when neoadjuvant therapy has not been used [47,57].

An open-label phase 2/3 trial highlighted the benefit of adjuvant chemotherapy for gastric adenocarcinoma [51]. In patients with stage II/III gastric or esophageal cancer, neoadjuvant therapy with epirubicin, cisplatin, and 5-fluorouracil exhibited superior outcomes compared with surgery alone, as evidenced by higher rates of progression-free and overall survival [58]. Furthermore, neoadjuvant therapy with 5-fluorouracil, leucovorin, oxaliplatin, and docetaxel showed better compliance and efficiency in terms of periods without tumor progression, overall survival, and complete the regression of pathologies compared with another perioperative regimen comprised of epirubicin, cisplatin, and 5-fluorouracil or epirubicin, cisplatin, and capecitabine [59].

The identification of tumor markers and the genetic profiling of cancers offer the possibility for personalized treatment regimens. In the case of GC, monoclonal antibodies and small-molecule inhibitors are used in targeted therapies. The elevated human epidermal growth factor receptor 2 (HER2) expression in GC makes this protein a suitable treatment target. HER2 belongs to the epidermal growth factor receptor (EGFR) family, which is involved in the tumor cell proliferation, migration, differentiation, apoptosis, and adhesion. Several studies have indicated the important role of HER2 in tumorigenesis in gastric cancer [60,61]. The overexpression of HER2 shows poor prognosis in gastric cancer patients [62]. The combination of trastuzumab (anti-HER2 monoclonal antibody) and chemotherapy showed improvements in overall survival and disease-free time compared with chemotherapy alone in advanced GC patients [63]. Another targeted therapeutic agent for GC is the anti-epidermal growth factor receptor (EGFR) monoclonal antibody nimotuzumab [64]. The combination of nimotuzumab with irinotecan improved the response rate and progression-free and overall survival in patients with advanced GC who were EGFR-overexpressed. Ramucirumab, a monoclonal antibody against vascular endothelial growth factor receptor (VEGFR)-2, prolonged median survival compared with a placebo in patients with advanced GC. Adding ramucirumab to paclitaxel prolonged the overall survival and periods without tumor progression compared with paclitaxel treatment alone in the same patients [65,66].

## 3. Nrf2 Inhibitors and Chemotherapy Drug Resistance

### 3.1. Anti-HER2 Drugs Resistance

HER2 has previously been reported to be elevated in 7 to 34% of GC patients [67,68]. The administration of anti-HER2 drugs including trastuzumab plus platinum-based chemotherapy could decrease the resistance to chemotherapy. However, resistance persists with trastuzumab plus platinum drug chemotherapy in tumors with amplified HER2 [69,70,71,72]. Gambardella and coworkers [61] investigated the mechanisms of primary and acquired resistance to the combination of trastuzumab and platinum-based chemotherapy [73]. Gambardella and coworkers [61] found that NRF2 amplified the resistance to anti-HER2 drugs through the PI3K/AKT/mTOR/RPS6 pathway, and that ribosomal protein S6 (RPS6) inhibition decreased NRF2 expression and restored sensitivity in HER2-amplified gastric cancer. 

The inhibition of RPS6 reduced the expression of Nrf2 and increased the sensitivity to chemotherapy in HER2-amplified GC cells. Yang and coworkers (2020) reported that brusatol, a modern Nrf2 inhibitor, showed anti-tumor properties, which were shown as decreased cancer cell growth by suppressing the Nrf2/HO-1 and HER2-AKT/ERK1/2 signaling in HER2-positive cancer cells (SK-OV-3 and BT-474 cells) [74]. 

It was found that brusatol exerted a synergistic effect with trastuzumab against HER2-positive cells by suppressing Nrf2/HO-1 and HER2-AKT/ERK1/2 signaling. 

**Figure 1:**
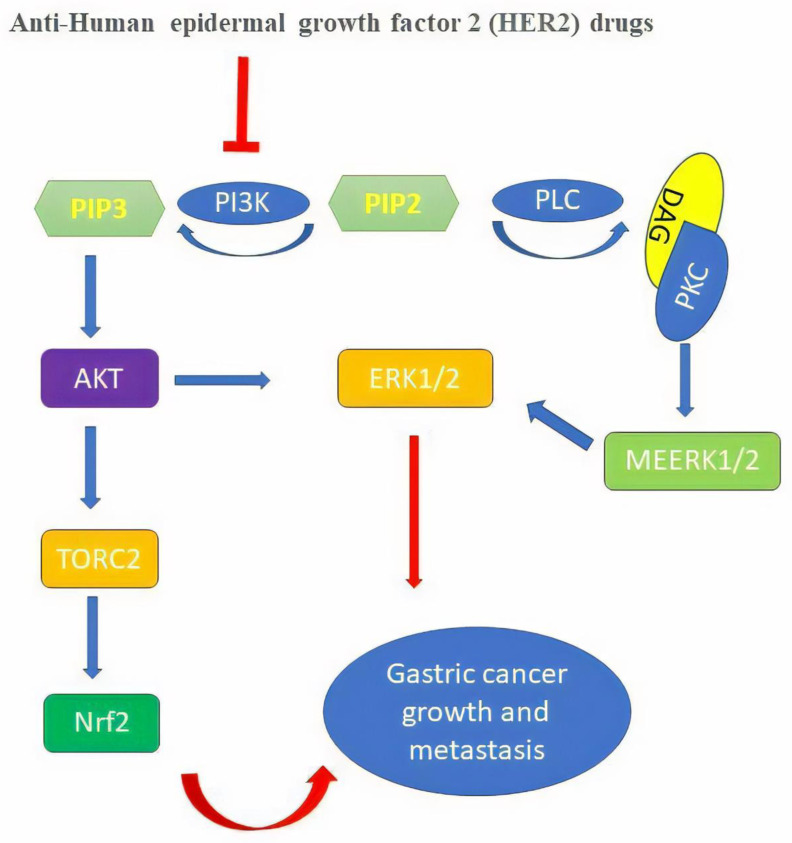
The inhibitory effect of anti-HER2 drugs on Nrf2 inhibition and gastric cancer prevention.

The combination of trastuzumab and brusatol significantly downregulated phosphorylation-HER2, as well as phosphorylation-ERK1/2 and phospho-AKT. However, the levels of the Nrf2 and HO-1 were reduced in the two HER2-positive cell lines by the combination therapy. Therefore, brusatol may increase the antitumor effect of trastuzumab via the inhibition of the Nrf2/HO-1 and HER2-AKT/ERK1/2 signaling pathway in HER2-positive cancer cells. Brusatol alone caused a moderate elevation of ROS production and apoptosis in BT-474 and SK-OV-3 cells. Meanwhile, trastuzumab plus brusatol significantly increased ROS accumulation and apoptosis when compared to brusatol or trastuzumab treatment alone in both BT-474 and SK-OV-3 cells [62]. Therefore, combining Nrf2 inhibition with anti-HER2 drugs is a suitable therapeutic strategy for the treatment of HER2-positive cancers (Figure 1).

### 3.2. 5-Fluorouracil Resistance

**Figure 2:**
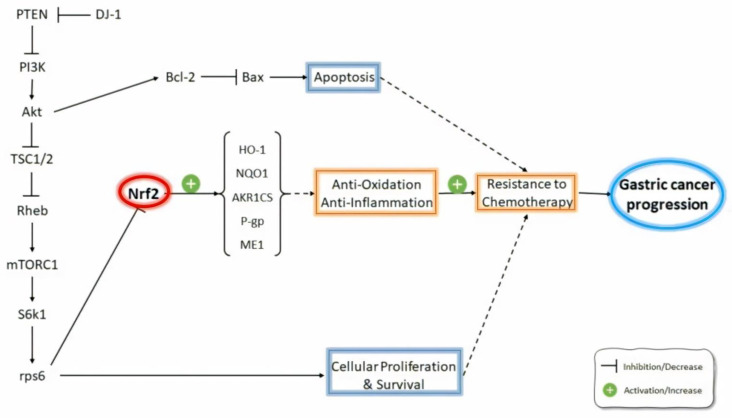
Role of Nrf2 pathways in gastric cancer.

Hu and coworkers [63] found that silencing the expression of Nrf2 decreased the resistance to fluorouracil (5-FU) and increased cell death in GC cell lines [75]. They also suggested that Nrf2 is an important predictive factor for 5-FU resistance in GC specimens. In addition, Pouremamali and coworkers [64] found that the effect of Nrf2 on malic enzyme-1 (ME-1) leads to the resistance of MKN-45 (MKN-45/DR) cells to 5-FU [76]. The ME enzyme exists in three forms in mammals, including ME-1 (cytosolic NADP+ dependent isoform), ME-2 (mitochondrial NAD+ dependent isoform), and ME-3 (mitochondrial NADP+ dependent isoform). ME-1 expression was induced in several human cancers [77]. GC cells with 5-FU resistance had decreased Nrf2 and ME-1 expressions and elevated MDR1 mRNA expression compared with 5-FU-susceptible cells. Thedministration of luteolin plus brusatol, two Nrf2 inhibitors, synergistically increased the cytotoxicity of 5-FU. MKN-45/DR cells incubated with luteolin plus brusatol elevated Nrf2 and ME-1 expression. They found that a combination of Nrf2 inhibitors is a suitable strategy for combating 5-FU resistance in GC patients (Figure 2).

### 3.3. Oxaliplatin Resistance

Oxaliplatin or Eloxatin is an alkylating chemotherapeutic agent that has high efficacy against gastrointestinal tract cancers in combination with 5-FU and folinic acid [78,79,80]. It has been reported that oxaliplatin produces cytotoxicity by forming platinum–DNA adducts that subsequently block DNA replication and induce cell death [81]. Resistance to oxaliplatin is caused by disturbed DNA adduct formation [82] and abnormalities in autophagy and apoptotic hypoxia following hypoxia-inducible factor-1a (HIF-1a) pathway stimulation [83,84]. Furthermore, the resistance of the human GC cell line TSGH-S3 (S3) to oxaliplatin may be due to the increased repairment of DNA and copper-transporting ATPase 1 (ATP7A) contents [85]. Furthermore, Chen and coworkers [85] investigated other resistance factors in S3 cells by using a gene array assay. The interleukin-6 (IL-6), aldo-keto reductase 1C1 (AKR1C1), and AKR1C3 genes are mostly up-regulated in S3 cells compared with TSGH parental cells. Since the AKR1Cs gene belongs to the ARE genes, Nrf2 inhibition decreased AKR1C1, AKR1C2, and AKR1C3 mRNA and reversed the oxaliplatin resistance in S3 cells (Figure 2). The findings indicated that the inhibition of the Nrf2/AKR1C pathway can reverse S3 cells’ resistance to oxaliplatin (Table 1).

### 3.4. Multidrug Resistance

It is well known that multi-drug resistance (MDR) is an important and serious problem for achieving success in chemotherapy. Interestingly, most ABC transporter genes, as the main regulator of MDR, are modulated via the Nrf2 signaling pathway [9,20]. Jeddi and co-workers [75] found increased expression and protein levels of Nrf2, as well as MDR1/P-gp, in GC patients [86]. The stimulation of P-gp (a drug efflux pump) was associated with Nrf2 induction, tumor size, and histological grade. Moreover, the inhibition of Nrf2 expression could increase the efficacy of chemotherapeutic agents by down-regulating the expression of P-gp in GC patients.

The overexpression of DJ-1, an oncogene product, is found in various malignant cancers, including GC [87,88]. Moreover, Zhu and co-workers [88] found elevated levels of DJ-1 protein expression in HeLa cells resistant to dihydroartemisinin (DHA), and the silencing of DJ-1 increased the DHA-sensitivity of HeLa cells. The findings indicated that DJ-1 has a major role in MDR in SGC7901 GC cells via the up-regulation of P-gp and Bcl-2 [89]. P-glycoprotein (P-gp) is a well-identified membrane transporter that is able to pump out drug molecules from the cancer cell and decrease chemotherapy efficiency. In cancer cells, the up-regulation of P-gp expression occurs as an adaptive response to combat cell death induced by chemotherapy [90]. P-gp is a well-identified membrane transporter with capability to efflux drug molecules out of the cancer cell leading to reduced efficiency of chemotherapy which is directly associated with elevated MDR in tumors. In addition, Bcl-2 overexpression can prevent the chemotherapy-induced expression of Fas/Fas ligand (FasL) and blocks chemotherapy-induced apoptosis through preventing the nuclear translocation of NFAT (nuclear factor of activated T lymphocytes, a transcription factor activated by microtubule damage). The FasL gene is not transcribed without NFAT nuclear translocation. Bcl-2 antagonizes drug-induced apoptosis by inhibiting calcineurin activation, blocking NFAT nuclear translocation and preventing FasL expression [91].

DJ-1 induces Nrf2/Keap1 uncoupling and thus can modulate Nrf2 activity [92,93]. However, DJ-1 cannot affect Nrf2 or Keap1 in a direct manner [92]. It has been found that Nrf2/Keap1 dissociation usually occurs following Nrf2 phosphorylation [94]. Nrf2 phosphorylation correlates with PI3K/Akt, PKC, etc. [95]. DJ-1 can bind to PTEN and prevents its activity, leading to the stimulation of PI3K/Akt [96,97]. A relation between the MDR of GC cells and the activation of PI3K/Akt has been found [98,99]. Qiu and co-workers (2020) showed that the DJ-1-related induction of MDR occurs through the stimulation of the PTEN/PI3K/Akt/Nrf2 signaling pathway, as well as P-gp and Bcl-2 up-regulation in SGC7901 GC cells [76] (Figure 2).

## 4. Natural Therapeutic Agents for Gastric Cancer and Nrf2

Cancer chemoprevention with natural agents has become a focus of attention in recent years. In this context, baicalin and its aglycone, baicalein, have been found to have anti-tumor effects. Baicalin is the main flavonoid found in several plants including *Scutellaria baicalensis Georgi, S. lateriflora L. S. galericulata*, *S. rivularis*, and *Oroxylum indicum (L.) Kurz* [100,101,102].

The anti-progressive effects of baicalein have been found in MGC-803, SGC-7901, SGC-7901/DDP, and HGC-27 GC cell lines [103]. Furthermore, adding baicalein to diamminedichloroplatinum (DDP) suppressed progression and elevated apoptosis and autophagy in SGC-7901 and SGC-7901/DDP GC cell lines. The modulatory effect of baicalein on Akt/mTOR and Nrf2/Keap 1 signaling affects SGC-7901/DDP GC cells’ sensitivity to chemotherapy. In addition, baicalein stimulated Akt/mTOR- and Nrf2/Keap1-mediated apoptosis and autophagy which subsequently increased the sensitivity of GC cells to DDP [95].

Baicalein significantly improved Nrf2 expression and decreased the Keap 1 level dose-dependently. This mechanism indicates the antioxidative activity of baicalein, and is not related to the stimulatory effect of baicalein on the DDP sensitivity of SGC-7901/DDP. However, baicalein down-regulated MDR1 expression in SGC-7901 and SGC-7901/DDP dose- and time-dependently [95].

Garlic (*Allium sativum* L.) is another natural compound with anti-cancer activity. Garlic consumption is accompanied with a decrease in the incidence of GC [104]. Diallyl trisulfide (DATS), an oil-soluble compound isolated from garlic, exerted antitumor effects [105]. Indeed, DATS acts as a chemotherapeutic agent through the induction of oxidative stress [106], cell cycle arrest, apoptosis, and also proliferation suppression [107], as well as tumor cell invasion and metastasis inhibition [108].

Jiang and co-workers [99] indicated the chemo-preventive effects of DATS in BGC-823 human GC cells, and DATS stimulated the anti-tumor effects of DDP in BGC-823 xenograft mice [109]. DATS could inhibit the viability and induced cell cycle arrest in the G2/M phase in BGC-823 cells in a dose-dependent manner. It was shown that the protective effects of DATS in BGC-823 cells were caused by activation of p38 and JNK/MAPK and the attenuation of the Nrf2/Akt signaling pathway. DATS reduced Akt phosphorylation in tumors, leading to a decrease in the Nrf2 levels both in vitro and in vivo. In anticancer therapy, the PI3K/Akt signaling pathway is involved in Nrf2 expression [101].

## 5. Biomarkers of Gastric Cancer and Nrf2

The lymphoid enhancer factor/T cell factor (LEF/TCF) family of transcription factors is localized in the nucleus. One member, transcription factor 7-like 1 (TCF7L1), is a DNA-specific binding protein that binds to β-catenin and regulates Wnt/β-catenin signaling [110]. TCF7L1 expression is up-regulated in malignant tumors and indicates a poor prognosis. It was found that in breast cancer and colorectal cancer, the elimination of TCF7L1 expression reduced tumor cell progression and metastasis [111]. In breast cancer and colorectal cancer, TCF7L1 regulates Wnt/β-catenin signaling [112]. Wnt signaling regulates proliferation and cell hemostasis in normal gastric mucosa; however, its activation can be found in GC development. The ability of TCF7L1 to regulate Wnt/β-catenin signaling implicates its role in GC prognosis.

In this context, Zhang and co-workers [103] showed that a higher expression of TCF7L1 is associated with a poorer prognosis in patients [113]. TCF7L1 was also found to negatively modulate the expression of Keap1, leading to Nrf2 activation. 

Peng and co-workers [104] investigated the association between high Nrf2 levels and the expression of NAD(P)H: quinone oxidoreductase 1 (NQO1) in chemotherapy-resistant GC patients [114]. It was found that Nrf2 and NQO1 expressions are indicators of developed GC. They also indicated that high Nrf2 expression in GC tissues directly correlates with the progression and metastatic features of GC, as well as resistance to 5-FU [106]. 

BDH2, also known as DHRS6, is a short-chain dehydrogenase/reductase [115], which is mostly expressed in the epithelial cells of the kidney, oral cavity, small intestine, and breast [116]. BDH2 independently indicates the poor prognosis of acute myeloid leukemia. BDH2 affects the progression of squamous cell carcinoma of the esophagus [117]. Liu and co-workers (2020) found that suppressed BDH2 expression was an indicator of tumor growth in GC tissue. BDH2 stimulated Keap1 and Nrf2 interactions, leading to a decrease in the Nrf2 translocation to the nucleus. The ubiquitination of Nrf2 blocked ARE activity, resulting in the inhibition of AktSer473 and mTORSer2448 phosphorylation, thereby changing the PI3K/Akt /mTOR pathway and ultimately reducing GC cell growth [109].

## 6. Conclusions

Anti-cancer modalities can use the Nrf2 signaling pathway as a therapeutic target. The role of Nrf2 in cancer is controversial, as it has protective effects in both normal and cancerous cells. In GC cells, the inhibition of Nrf2 increased their sensitivity towards chemotherapeutic agents. Accordingly, Nrf2 inhibitors abolished the resistance of GC cells to several anti-HER2 drugs, 5-FU, and oxaliplatin. The inhibition of Nrf2 expression can be achieved by inhibiting the RPS6, PTEN/PI3K/Akt/Nrf2, PI3K/AKT/mTOR/RPS6, Nrf2/HO-1, and HER2-AKT/ERK1/2 pathways in GC. In addition, the inhibition of the Nrf2/AKR1C pathway reverses resistance in S3 cells. However, most experimental studies indicated the proactive effect of natural antioxidants against cancer through the Nrf2 signaling pathway. In this context, baicalin, DATS, and luteolin attenuated Nrf2 signaling and increased the sensitivity towards chemotherapeutic agents in different GC cell lines. There are several biomarkers related to the high expression of Nrf2, which indicates the poor prognosis of GC. In this context, TCF7L1, NQO1, and BDH2 can act as biomarkers for the poor prognosis of GC, which also indicate Nrf2 induction. It has been suggested that the evaluation of these biomarkers may be effective in providing an appropriate therapeutic approach for the management of GC. Altogether, numerous scientific reports indicate the efficacy of targeting Nrf2 in various diseases. In addition, there is evidence of the beneficial effect of Nrf2 activity and the suppressive role of Nrf2 inhibitors against tumor progression in human cancers. However, we should clearly inform that contradictory activity of Nrf2 in tumor progression has been found in recent years.

## Figures and Tables

**Table 1:** Modulation of gastric cancer through the Nrf2 pathway.

Authors	Investigation Aims	Subjects	Possible Roles of Nrf2
Gambardella et al. [73]	Mechanisms of primary and acquired resistance to the combination of trastuzumab and platinum-based chemotherapy	Lapatinib- and trastuzumab-resistant clones derived from two different HER2-amplified gastric cancer cell lines	NRF2 amplified the resistance to anti-HER2 drugs through the PI3K/AKT/mTOR/RPS6 pathway; RPS6 inhibition decreased NRF2 expression and restored sensitivity in HER2-amplified gastric cancer.
Yang et al. [74]	Efficiency of combination chemotherapy with trastuzumab and brusatol	HER2-positive SK-OV-3 and BT-474 cancer cells	Brusatol increased the antitumor activity of trastuzumab through the inhibition of the Nrf2/HO-1 and HER2-AKT/ERK1/2 signaling pathways.
Hu et al. [75]	Nrf2 protein expression in gastric cancer specimens + association between Nrf2 expression and 5-FU resistance	Samples from GC patients with gastrectomy and from GC patients who received first-line combination chemotherapy of either A) docetaxel, cisplatin, and 5-FU or B) S-1 plus cisplatin	Nrf2 was an independent prognostic factor in GC and caused resistance to the chemotherapeutic drug 5-FU in GC cells.
Pouremamali et al. [76]	The role of Nrf2 and its downstream target malic enzyme-1 (ME-1) in the resistance to 5-FU chemotherapy	Resistant MKN-45 (MKN-45/DR) cell line	In resistant cells, decreased Nrf2 and ME-1 expression levels increased MDR1 mRNA levels; the inhibition of Nrf2 with luteolin and brusatol increased the effects on 5-FU-induced cytotoxicity.
Jeddi et al. [75]	Association of Nrf2 and MDR1/P-gp in GC patients	Endoscopic biopsy samples from GC patients	Higher Nrf2 expression in GC patients compared with non-GC individuals; the induction of P-gp was associated with Nrf2 overexpression; there was correlation between Nrf2 overexpression and tumor size, histological grade, lymph node, and distant metastasis; P-gp up-regulation was associated with the histological grade and tumor size; the inhibition of Nrf2 expression improved the efficacy of chemotherapeutic agents for GC patients by the downregulation of P-gp expression.
Chen et al. [85]	Mechanisms of oxaliplatin resistance	Human gastric carcinoma cell line TSGH-S3 (S3)	The activation of Nrf2/AKR1C axis contributed to oxaliplatin resistance as the suppression of Nrf2 expression decreased the levels of AKR1C1, AKR1C2, and AKR1C3 and reversed oxaliplatin resistance in S3 cells.
Qiu et al. [86]	Mechanisms of DJ-1-induced multidrug resistance (MDR)	SGC7901 + MDR variant SGC7901/VCR gastric cancer cells	DJ-1 mediated the development of MDR in SGC7901 gastric cancer cells through inhibiting PTEN, activating the PI3k/Akt pathway and consequently resulting in Nrf2 activation, thereby inducing Nrf2-dependent P-glycoprotein (P-gp) and Bcl-2 expressions.

## Abbreviations

5-FU	5-fluorouracil
AKR	Aldo-keto reductase
AREs	Antioxidant responsive elements
BCL2	B-cell lymphoma 2
BDH2	3-Hydroxybutyrate dehydrogenase 2
DATS	Diallyl trisulfide
DHA	Dihydroartemisinin
DNA	Deoxyribonucleic acid
EBV	Epstein–Barr virus
EGFR	Anti-epidermal growth factor receptor
GC	Gastric cancer
GCL	Glutamate–cysteine ligase
GRIM	Retinoid-IFN-induced mortality
HER2	Human epidermal growth factor receptor 2
HIF-1a	Hypoxia-inducible factor-1a
HO-1	Heme oxygenase-1
IL	Interleukin
Keap1	Kelch-like ECH-associated protein 1
LEF	Lymphoid enhancer factor
MAF	Musculoaponeurotic fibrosarcoma
MARE	MAF recognition elements
MDR	Multidrug resistance
ME	Malic enzyme
NAD	Nicotinamide adenine dinucleotide
NQO1	Quinone oxidoreductase 1
Nrf2	Nuclear factor erythroid 2-related factor 2
P-gp	P-glycoprotein 1
RNA	Ribonucleic acid
ROS	Reactive oxygen species
RPS6	Ribosomal protein S6
TCF	T cell factor
VEGF	Vascular endothelial growth factor
VEGFR	Anti-vascular endothelial growth factor receptor
